# The Effects of Excess Co on the Phase Composition and Thermoelectric Properties of Half-Heusler NbCoSb

**DOI:** 10.3390/ma11050773

**Published:** 2018-05-11

**Authors:** Lihong Huang, Junchen Wang, Xi Chen, Ran He, Jing Shuai, Jianjun Zhang, Qinyong Zhang, Zhifeng Ren

**Affiliations:** 1 Key Laboratory of Fluid and Power Machinery of Ministry of Education, School of Materials Science & Engineering, Xihua University, Chengdu 610039, China; huang.lihong@foxmail.com (L.H.); junchenwang123@sina.com (J.W.); 15183544928@163.com (X.C.); bohr123@163.com (Q.Z.); 2 Institute for Metallic Materials, IFW-Dresden, 01069 Dresden, Germany; zhizhishangshan@gmail.com; 3 Department of Physics and TcSUH, University of Houston, Houston, TX 77204, USA; jshuai@uh.edu

**Keywords:** half-Heusler, NbCoSb, excess Co, thermoelectric performance

## Abstract

NbCoSb with nominal 19 valence electrons, and is supposed to be metallic, has recently been reported to also exhibit the thermoelectric properties of a heavily doped n-type semiconductor. In this study, we prepared Co-rich NbCo_1+*x*_Sb samples (*x* = 0, 0.2, 0.3, 0.4, 0.5), and their phase compositions, microstructures and thermoelectric properties were investigated. The Seebeck coefficient increased a great deal with increasing *x*, due to decreasing carrier concentration, and the total thermal conductivity reduced mainly because of declining *κ*_e_. Finally, a peak thermoelectric figure of merit, *ZT*, was about 0.46 for NbCo_1.3_Sb at 973 K. This enhancement was mainly attributed to the reduction of electric thermal conductivity and the increase of Seebeck coefficient. The excess Co had effects on the carrier concentration, deformation potential *E*_def_ and DOS effective mass m^*^. Adding an excessive amount of Co leads to a very high *E*_def_, which was detrimental for transport characteristics.

## 1. Introduction

Thermoelectric (TE) materials can directly convert waste heat into electricity in an environmentally-friendly way and have received a great deal of attention owing to global energy sources and environmental challenges [1]. The conversion efficiency of a TE material is determined by the dimensionless figure of merit *ZT* = *S*^2^*σT*/(*κ*_e_ *+ κ*_L_), where *S*, *σ*, *T*, *κ*_e,_ and *κ*_L_ are the Seebeck coefficient, electrical conductivity, absolute temperature, and electronic and lattice components of the thermal conductivity, respectively. It is hard to achieve a high *ZT* by simply improving one of these parameters, because *S*, *σ, κ*_e_ are strongly coupled with each other [2]. Hence, the optimization of carrier concentrations is an essential requirement [3]. 

Usually, an ideal thermoelectric material should have a glass-like heat conduction of phonons and crystal-like electrical conduction of charge carriers. This strategy is referred to as “phonon-glass electron-crystal”, and was first introduced by Slack [4]. To improve phonon transport properties, various approaches have been used to enhance phonon scattering and decrease *κ*_L_, such as introducing secondary phase [5], nanoscale crystalline grain [6] and point defect [7]. To enhance electron transport properties, a series of band structure engineering approaches have been developed to tune the power factor (*S^2^σ*) by optimizing carrier concentration [8] or energy resonant doping [9].

Recently, particular attention has been paid to half-Heusler (HH) compounds, due to their high-temperature stability, good mechanical robustness, and good TE performance, which are of paramount importance for practical applications. Typical semiconducting HH compounds with 18 valence electrons per unit cell, ZrCoSb, ZrNiSn, TiNiSn, NbFeSb, and their alloys have been intensively investigated as promising medium–high temperature TE materials [10,11,12,13,14,15,16]. Additionally, an exciting *ZT* of about 1.5 at 1200 K has been realized in p-type NbFeSb alloys [17,18]. Surprisingly, our previous work showed that NbCoSb with nominal 19 valence electrons, which is supposed to be metallic, exhibited fairly good n-type TE performance and achieved a peak *ZT* of ~0.4 at 973 K [19]. The experimental results indicated that NbCoSb is a heavily doped n-type semiconductor with high carrier concentration. 

From our previous work, we know that NbCoSb has a comparatively low Seebeck coefficient and a higher thermal conductivity. According to Chai’s report [20,21], *ZT* of ZrNi_1.1_Sn was approx. 40% higher compared to ZrNiSn, due to the secondary phase in the matrix introduced by excess Ni. It provided a great basis for phonon-scattering centers, leading to an improved Seebeck coefficient and a reduced thermal conductivity [20,21]. Inspired by this feature, this work aimed at qualitatively investigating the effects of excess Co on the microstructures and thermoelectric properties of HH alloy NbCoSb. The Co-rich samples with nominal compositions of NbCo_1+*x*_Sb (*x* = 0, 0.2, 0.3, 0.4, 0.5) were prepared through arc melting, following by ball milling and hot pressing. The evaluation and comparison of phase composition and TE properties of NbCo_1+*x*_Sb were performed. Additionally, the electrical transport characteristics of Co-rich NbCoSb materials were also analyzed using the single parabolic band (SPB) model.

## 2. Experimental Procedures

A series of Co-rich NbCoSb samples were prepared by arc melting followed by ball milling and hot pressing. High purity niobium slugs (Nb, 99.99%, Alfa Aesar, Ward Hill, MA, USA), cobalt pieces (Co, 99.95%, Alfa Aesar), and antimony rods (Sb, 99.8%, Alfa Aesar) were weighed according to the composition of NbCo_1+*x*_Sb (*x* = 0, 0.2, 0.3, 0.4, 0.5). For all samples, 5 at% more Sb was added in order to compensate the loss of Sb during arc melting. In order to get homogeneous ingots, metallic raw materials were arc-melted five times reversely under argon protection. The obtained ingots were ball milled (SPEX 8000M Mixer/Mill, Metuchen, NJ, USA) for 7 hours under argon protection. Thereafter, the ball-milled powders were loaded into a graphite die and sintered by direct current-induced hot pressing at 1273 K for 2 min under 77 MPa to get pellet samples. The thickness of the as-hot-pressed samples was about 2 mm.

The thermal conductivity *κ* = *dDC_p_* was calculated using the measured density (*d*) by Archimedes method, thermal diffusivity (*D*) by laser flash method (LFA 457, Netzsch), and specific heat capacity (*C_p_*) by differential scanning calorimetry (DSC 404 C, Netzsch, Selb, Germany). Bar-shaped samples of about 2 mm × 2 mm × 10 mm were used for measuring the electrical conductivity and Seebeck coefficient simultaneously from 300 to 973 K (ZEM-3, ULVAC Riko, Chigasaki, Japan). The Hall coefficient (*R*_H_) was measured at room temperature by a commercial system (PPMS D060, Quantum Design, San Diego, CA, USA) using a four-probe configuration and the Van der Pauw method, with a magnetic field of 3.0 T and an electrical current of 8 mA. The carrier concentration (*n*) was calculated by *n* = 1/(*eR*_H_), and the carrier mobility (*μ*) was estimated by *σ* = *neμ*. The properties of the samples were isotropic, due to their small grain size and random crystal orientation.

The phase structure of the samples was observed using XRD on a PANalytical X’Pert Pro diffractometer (Malvern Panalytical, Almelo, Holland) and Cu Kα radiation. The fresh section of the hot-pressed sample NbCo_1.3_Sb was studied via SEM (JEOL 6330F, Deerbrook, WI, USA) to show the particle size.

## 3. Results and Discussion

**Figure 1:**
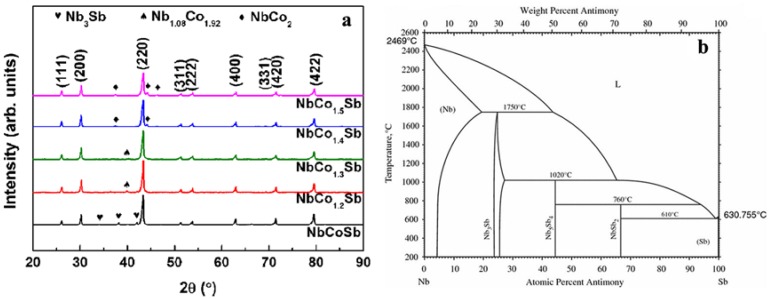
XRD patterns of bulk NbCo_1+*x*_Sb (*x* = 0, 0.2, 0.3, 0.4, 0.5) samples (**a**) the binary phase diagram of Nb and Sb (**b**).

Figure 1a shows the XRD patterns of hot-pressed bulk samples NbCo_1+*x*_Sb (*x* = 0, 0.2, 0.3, 0.4, 0.5) which can be classified as cubic MgAgAs-type crystal structure (JCPDS 51-1247, *a* = 0.5897 nm). The dominant phase of all samples was the half–Heusler phase of NbCoSb, while a small number of secondary phases such as Nb_3_Sb, Nb_1.08_Co_1.92_, and NbCo_2_ were observed following the increasing content of excess Co, respectively. Based on the binary phase diagram of Nb and Sb, Nb_3_Sb may be formed, due to the peritectic reaction during the cooling process (*x* = 0), as shown in Figure 1b. With a small amount of excess Co (*x* = 0.2, 0.3), the secondary phases Nb_3_Sb disappeared. However, another secondary phase Nb_1.08_Co_1.92_ was introduced in a small amount. A new secondary phase NbCo_2_ was observed for the Co-rich sample NbCo_1.4_Sb, with increased content of excess Co. Even more NbCo_2_ appeared for NbCo_1.5_Sb. These phenomena indicate a scale of Co solubility in the structure. However, when *x* exceeded 0.3, it precipitated out the other second phase of NbCo_2_.

Table 1 shows the lattice parameter, theoretical, experimental, and relative density of NbCo_1+*x*_Sb samples. The lattice parameter increased with increasing *x*, mainly due to the solid solution of excess Co, leading to lattice expansion of HH phase. The theoretical density was calculated by *d*_cal_ = ∑*n*_i_M_i_/(*a*^3^*N_A_*), where *n*_i_ is the number of atoms of Nb, Co and Sb per unit cell, M_i_ the corresponding atomic mass of each element, *a* the lattice parameter, and *N_A_* the Avogadro constant (6.023 × 10^23^ mol^−1^). The relative density was more than 90% for *x* = 0, 0.2, 0.3 and approx. 85–86% for *x* = 0.4 and 0.5. The low relative density of the NbCo_1.4_Sb and NbCo_1.5_Sb samples may be due to inaccurate estimation of the theoretical density. The additional Co did not fully enter the lattice, but form the second phase, while we ignored this in our calculations.

**Figure 2:**
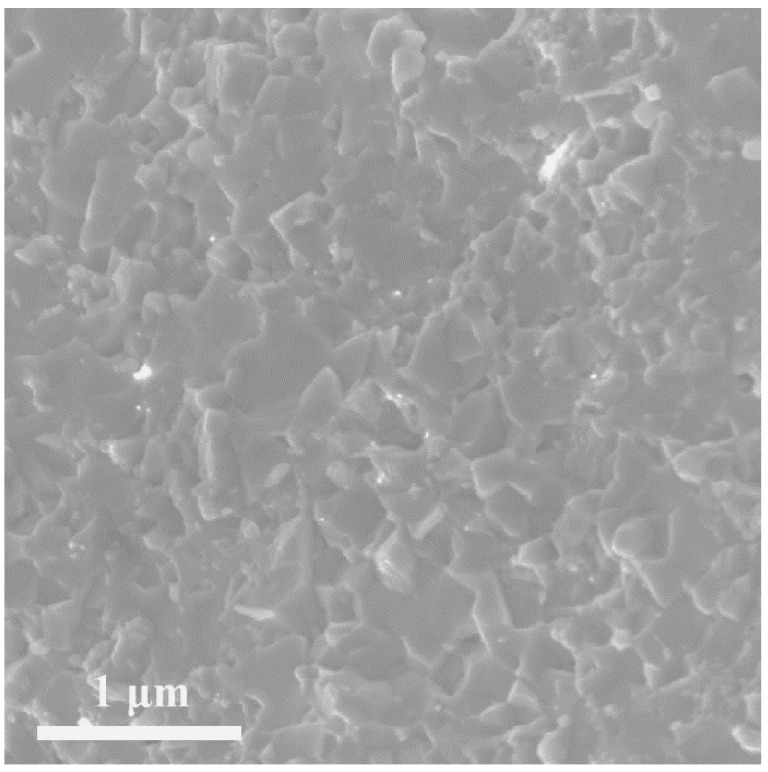
SEM image of hot pressed sample NbCo_1.3_Sb.

As shown in Figure 2, there were no pores in our hot-pressed sample NbCo_1.3_Sb, indicating a high relative density. Moreover, the grainsize of the sample was approx. several hundreds of nanometers.

**Figure 3:**
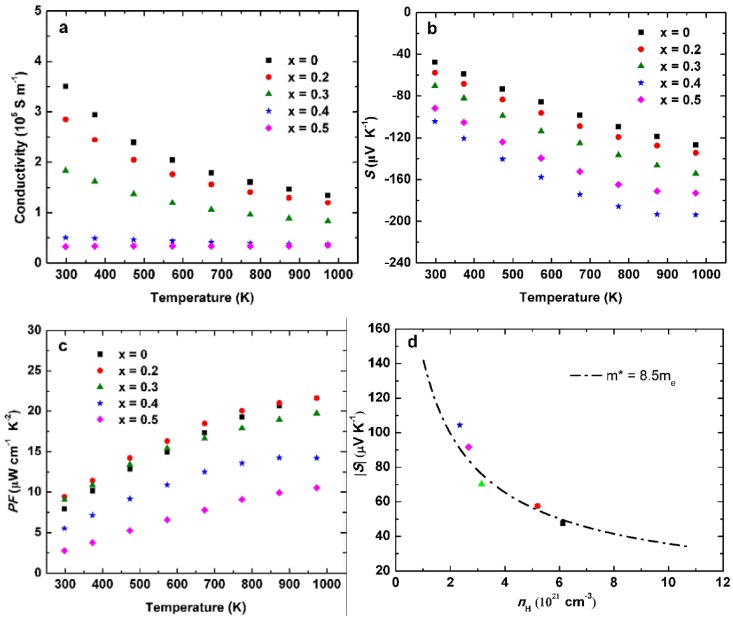
(**a**) Temperature-dependent electrical conductivity; (**b**) Seebeck coefficient; (**c**) power factor; and (**d**) and Pisarenko plot for NbCo_1+*x*_Sb.

Figure 3 shows the electrical properties as a function of temperature for NbCo_1+*x*_Sb. The electrical conductivity of NbCo_1+*x*_Sb decreased with increasing temperature for *x* = 0, 0.2, 0.3, indicating the characteristics of metal (Figure 3a). However, the trend of decreasing electrical conductivity with increasing temperature was not determinable for NbCo_1.4_Sb and NbCo_1.5_Sb. Apparently, the additional Co reduced the electrical conductivity, and the decrease was not present for *x* = 0.5. For NbCo_1.3_Sb, the electrical conductivity was approx. 1.83 × 10^5^ S m^−1^ at room temperature and reached approx. 0.83 × 10^5^ S m^−1^ at 973 K. As shown in Figure 3b, the negative Seebeck coefficients of all samples indicated a n-type conduction behavior. The Seebeck coefficient of NbCo_1.3_Sb was approx. −70 μV K^−1^ at room temperature and ~ −154 μV K^−1^ at 973 K. The Seebeck coefficient enhanced gradually with increasing excess Co content, which could partly be explained by a decreasing Hall carrier concentration (Table 2). The power factor for NbCo_1.3_Sb was ~9 × 10^−4^ W m^−1^ K^−2^ at room temperature and ~20 × 10^−4^ W m^−1^ K^−2^ at 973 K (Figure 3c). The power factor was maintained or decreased after adding excess Co, especially for NbCo_1.4_Sb and NbCo_1.5_Sb, due to their low electrical conductivity. Pisarenko plot of |*S*| versus carrier concentration (*n*) at room temperature was calculated based on Equations (1)–(6) using a SPB model. The results are shown in Figure 3d [22,23].

(1) $$S=\pm \frac{k_{B}}{e}\left(\frac{\left(r+5/2\right)F_{r+3/2}\left(\eta \right)}{\left(r+3/2\right)F_{r+1/2}\left(\eta \right)}-\eta \right)$$

(2) $$F_{n}\left(\eta \right)=\int_{0}^{\infty }\frac{x^{n}}{1+e^{x-\eta }}dx$$

(3) $$L={\left(\frac{k_{B}}{e}\right)}^{2}\left[\frac{\left(r+7/2\right)F_{r+5/2}\left(\eta \right)}{\left(r+3/2\right)F_{r+1/2}\left(\eta \right)}-{\left(\frac{\left(r+5/2\right)F_{r+3/2}\left(\eta \right)}{\left(r+3/2\right)F_{r+1/2}\left(\eta \right)}\right)}^{2}\right]$$

(4) $$r_{H}=\frac{3}{2}F_{1/2}\left(\eta \right)\frac{\left(3/2+2r\right)F_{2r+1/2}\left(\eta \right)}{{\left(3/2+r\right)}^{2}F_{r+1/2}^{2}\left(\eta \right)}$$

(5) $$n=\frac{4\pi {\left(2m^{*}k_{B}T\right)}^{3/2}F_{1/2}\left(\eta \right)}{h^{3}r_{H}}$$

(6) $$m^{*}=\frac{h^{2}}{2k_{B}T}{\left[\frac{n\times r_{H}}{4\pi F_{1/2}\left(\eta \right)}\right]}^{2/3}$$

where *F_n_*(*η*) is the *n*th order Fermi integral, *η* the reduced Fermi energy, *e* the electron charge, *r* the scattering factor, *k_B_* the Boltzmann’s constant, *h* the Plank’s constant, *r_H_* the Hall factor, and *x* the variable of integration. *r* = −1/2 was used for the acoustic phonon scattering mechanism. Combining the experimental carrier concentrations and Seebeck coefficients, a density of states (DOS) effective mass m^*^ ~8.5 *m*_e_ was derived for Co-rich samples NbCo_1+*x*_Sb. Therefore, the improvements of Seebeck coefficients for Co-rich samples could be explained by their relatively high DOS effective mass m^*^ and reduced carrier concentration.

Table 2 shows the electrical transport properties of all samples at room temperature. Hall coefficients showed negative values, indicating n-type conduction with electrons as major carriers, which was consistent with the negative values of the Seebeck coefficients. Obviously, the carrier concentration decreased with increasing *x*. The mobility of the Co-rich samples was largely decreased for *x* = 0.4 and 0.5, which may be due to their enhanced effective mass and existence of secondary phases. 

The deformation potential *E_def_* specifically characterizes the scattering effects of lattice vibration on carriers, namely the coupling effects between phonons and electrons. *E_def_* can be roughly estimated by Equation (7) when acoustic phonon scattering is dominant [24,25].

(7) $$E_{def}^{2}=\frac{F_{0}\left(\eta \right)}{2F_{1/2}\left(\eta \right)}\frac{2\sqrt{2}e\pi h^{4}}{3{\left(k_{B}T\right)}^{3/2}}\frac{dv_{l}^{2}N_{v}^{5/3}}{\mu_{H}{\left(m^{*}\right)}^{5/2}}$$

where *d* is the density (approx. 8.8 g cm^−3^), *v_l_* the longitudinal velocity of sound (approx. 5488 m s^−1^), *µ*_H_ the drift mobility (*µ*_H_ = *µ*/*r*_H_), and *Nv* the number of degenerate carrier pockets. The deformation potential *E_def_* of NbCo_1.5_Sb was found to be 41.38 eV (Table 2), a relatively large value, being detrimental for electrical transport properties. It was much higher than the one of NbCoSb_0.8_Sn_0.2_ (approx. 21 eV) [26].

**Figure 4:**
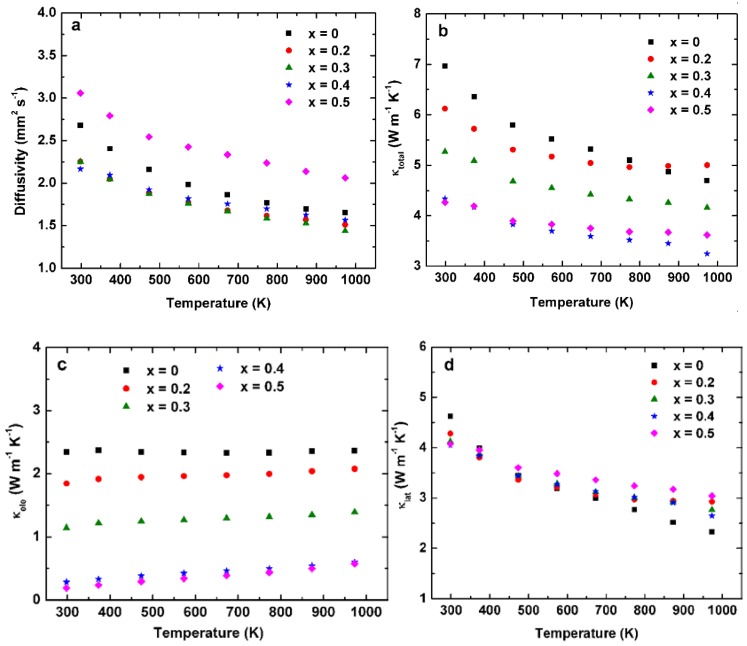
(**a**) Temperature-dependent thermal diffusivity; (**b**) total thermal conductivity; (**c**) electronic thermal conductivity; and (**d**) lattice thermal conductivity for NbCo_1+*x*_Sb.

Figure 4 shows the thermal transport properties of NbCo_1+*x*_Sb. Obviously, diffusivity showed a negative linear relationship with temperature and slightly decreased after adding excess Co except for *x* = 0.5 (Figure 4a). As shown in Figure 4b, the total thermal conductivity *κ* of NbCo_1+*x*_Sb decreased with increasing *x*, primarily due to reduced electronic thermal conductivities. Besides, *κ* decreased during the whole test temperature range, because of the Umklapp scattering process, indicating no bipolar effect for this system. The total thermal conductivity is the sum of lattice thermal conductivity *κ*_L_ and electronic thermal conductivity *κ*_e_, assuming negligible bipolar effect. The Wiedemann–Franz relation *κ*_e_ = *LσT*—where *L* is the Lorenz number which can be estimated using SPB model by acoustic phonon scattering using Equations (1)–(3)—combines the measured Seebeck coefficients. The calculated Lorenz constant was approx. 2.0 × 10^−8^ W Ω K^−2^ for NbCo_1.3_Sb at room temperature. *κ*_L_ could be calculated by subtraction of *κ*_e_ from *κ*. As shown in Figure 4c,d, *κ*_L_ increased slightly for Co-rich samples, while *κ*_e_ dropped steadily over the whole measured temperature range. Here, the reduction of *κ*_e_ can be primarily ascribed to both the reduction of *L* and *σ*. The obvious decrease of *κ*_e_ happened due to reduced carrier concentration, contributing to a reduced Lorenz constant. Typically, *κ*_e_ at room temperature decreased more than 70% from 2.34 W m^−1^ K^−1^ for NbCoSb to 0.19 W m^−1^ K^−1^ for NbCo_1.5_Sb. Moreover, the slight increase of *κ*_L_ may be due to weakened electron-phonon scattering, which became weaker for Co-rich samples [27]. It might also related to the existence of impurity phases Nb_1.08_Co_1.92_ and NbCo_2_, or other possible phonon scattering. 

Furthermore, we estimated the minimum lattice thermal conductivity (*κ_min_*) of NbCoSb using Cahill’s method, assuming the phonon’s mean free path is half of the phonon’s wavelength [28]:

(8) $$\kappa_{\min}=\frac{1}{2}{\left(\frac{\pi }{6}\right)}^{1/3}k_{B}V^{-2/3}\left(2v_{t}+v_{l}\right)$$

where *V*, *ν_t_*, and *ν_l_* are average volume per atom, transversal and longitudinal wave velocity (both measured at room temperature; *ν_t_* = 3059 m s^−1^, *ν_l_* = 5488 m s^−1^), respectively. In general, *κ* was found to be larger than the estimated *κ_min_* ≈ 0.9 W m^−1^ K^−1^. It seems that there is a large potential to improve the *ZT* values by further reducing the lattice thermal conductivity of NbCoSb based TE materials, definitely, further efforts are required to explore new useful ways to enhance the thermoelectric performance.

**Figure 5:**
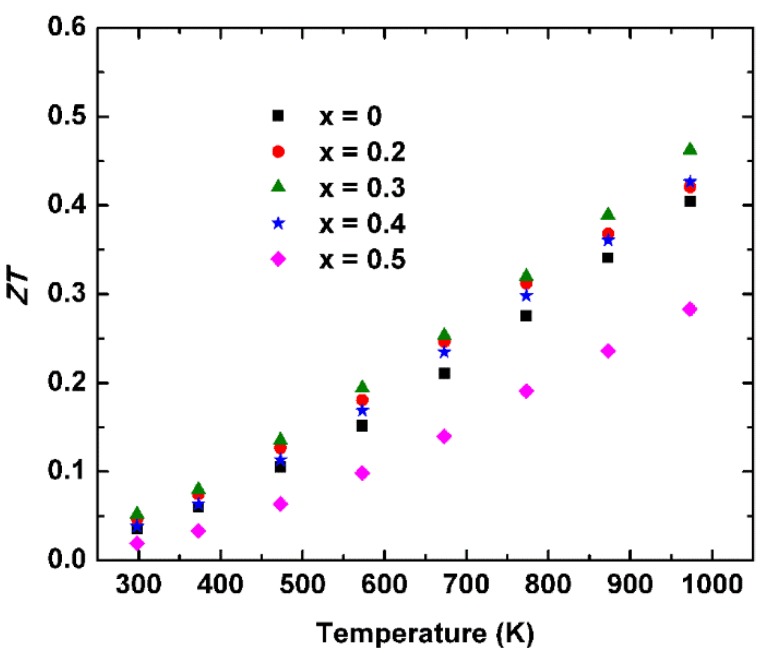
Temperature dependent *ZT* values of half-Heusler compounds NbCo_1+*x*_Sb (*x* = 0, 0.2, 0.3, 0.4, 0.5).

Combining all transport properties, the figure of merit *ZT* was calculated as a function of temperature as shown in Figure 5. The peak *ZT* of 0.46 was obtained at 973 K for sample NbCo_1.3_Sb. A slight increase of *ZT* should contribute to decreased thermal conductivity and a moderate *PF*.

## 4. Conclusions

In summary, Co-rich samples NbCo_1+*x*_Sb (*x* = 0, 0.2, 0.3, 0.4, 0.5) were synthesized and characterized. A small change in lattice parameter indicated a certain degree of Co solution into half-Heusler matrix. Adding excess Co eliminated the secondary phase of Nb_3_Sb, while other impurity phases such as Nb_1.08_Co_1.92_ and NbCo_2_ appeared with more excess Co. With increasing *x*, the Seebeck coefficient increased a lot due to decreasing carrier concentration; total thermal conductivity decreased mainly because of declining *κ*_e_. Adding an excessive amount of Co leads to a very high deformation potential *E*_def_, such as NbCo_1.5_Sb (41.38 eV), which is detrimental for transport characteristics. Finally, a maximum *ZT* value of approx. 0.46 for NbCo_1.3_Sb was obtained at 973 K.

## Figures and Tables

**Table 1:** Lattice parameter, theoretical, experimental, and relative density of NbCo_1+*x*_Sb (*x* = 0, 0.2, 0.3, 0.4, 0.5) samples.

Nominal Composition	Lattice Parameter (nm)	Density (g cm^−3^)		Relative Density (%)
		Theoretical	Experimental	
NbCoSb	0.5890	8.893	8.329	93.66
NbCo_1.2_Sb	0.5893	9.262	8.345	90.10
NbCo_1.3_Sb	0.5895	9.443	8.637	91.46
NbCo_1.4_Sb	0.5896	9.634	8.310	86.26
NbCo_1.5_Sb	0.5897	9.817	8.342	84.98

**Table 2:** Hall coefficient, carrier concentration, Hall mobility, effective mass, and deformation potential of NbCo_1+*x*_Sb (*x* = 0, 0.2, 0.3, 0.4, 0.5) at room temperature.

Nominal Composition	*R*_H_	*n*_H_ (10^21^ cm^−3^)	*μ*_H_ (cm^2^ V^−1^ s^−1^)	*E*_def_ (eV)
NbCoSb	−1.03	6.04	3.62	18.86
NbCo_1.2_Sb	−1.21	5.16	3.45	18.21
NbCo_1.3_Sb	−2.01	3.11	3.68	21.93
NbCo_1.4_Sb	−2.66	2.35	1.34	30.71
NbCo_1.5_Sb	−2.27	2.75	0.74	41.38
